# Variants of ESR1, APOE, LPL and IL-6 loci in young healthy subjects: association with lipid status and obesity

**DOI:** 10.1186/1756-0500-2-203

**Published:** 2009-10-05

**Authors:** Jadranka Sertic, Ljiljana Juricic, Hana Ljubic, Tamara Bozina, Jasna Lovric, Jasenka Markeljevic, Bojan Jelakovic, Marijan Merkler, Zeljko Reiner

**Affiliations:** 1 Clinical Institute of Laboratory Diagnosis, Zagreb University Hospital Centre, Zagreb, Croatia; 2 Department of Chemistry, Biochemistry and Clinical Biochemistry, School of Medicine, University of Zagreb, Zagreb, Croatia; 3 Department of Internal Medicine, Zagreb University Hospital Centre, Zagreb, Croatia; 4 Division of Nephrology and Arterial Hypertension, Department of Internal Medicine, Zagreb University Hospital Centre, Zagreb, Croatia; 5 Division of Metabolic Diseases, Department of Internal Medicine, Zagreb University Hospital Centre, Zagreb, Croatia

## Abstract

**Findings:**

BMI was increased (>25) in 22% of young healthy subjects. Increased cholesterol values (>5.0 mmol/L) were found in 23% of subjects, LDL-C (>3.0 mmol/L) in 23%, triglycerides (>1.7 mmol/L) in 11% of subjects. We found statistically significant differences in subjects' weight (p = 0.015), BMI (p = 0.023), and waist-hip ratio (WHR) (p = 0.015) in regard to their diet type; subjects with Mediterranean diet had the lowest values compared to those on continental and mixed diet. Significant associations were found for: LPL genetic polymorphic variant and abdominal obesity (p = 0.013), APO epsilon4 allele and hypercholesterolemia (p = 0.003), and ESR1-TA long allele and hypercholesterolemia (p = 0.011).

**Background:**

Human obesity is a multifactorial syndrome influenced also by genetic factors. Among gene variants found to be involved in body weight regulation and development of obesity, particular attention has been paid to polymorphisms in genes associated with obesity-related metabolic disorders. We explored the association of genetic polymorphisms of: estrogen receptor alpha (ESR1-TA repeats); interleukin-6 (IL-6 G-174C); apolipoprotein E (APO epsilon2, epsilon3, epsilon4); lipoprotein lipase Pvu II (LPL P+/-), with clinical variables: gender, age, body mass index (BMI), diet type and biological variables: triglycerides, cholesterol, HDL-C, LDL-C, CRP, homocysteine, urate, and glucose in 105 healthy young subjects (20-35 yrs) of Croatian origin.

**Methods:**

Genotyping of IL-6, LPL was performed by PCR-RFLP, of APOE by real-time PCR, and of ESR1 by PCR and capillary electrophoresis. Association analyses were performed of alleles and genotypes with biological variables.

**Conclusion:**

ESR-1, LPL, and APO E genetic polymorphic variants could represent predictive genetic risk markers for obesity-related metabolic disorders in young healthy subjects. Mediterranean type of diet is also an important protective factor against abdominal obesity.

## Background

Obesity has become an extensive public health concern because its prevalence has increased to epidemic proportions.

Many gene variants are involved in development of obesity and body weight regulation [1-3].

The most frequently investigated gene polymorphisms are APOE, adiponectin (ADIPOQ), angiotensin-converting enzyme (ACE), methyltetrahydrofolate reductase (MTHFR), IL-6, and estrogen receptors α and β (ESR1, ESR2) [4-7].

Our study was focused on ESR1 (L-long TA repeats; S-short TA repeats), APOE, IL-6 and LPL in young healthy population. Estrogen appears to be involved in protection against obesity and regulation of food intake. ESR1 is expressed mainly by adipocytes and it lowers the lipolytic response in subcutaneous fat by increasing the number of antilipolytic adrenoreceptors. BMI, waist circumference and HDL cholesterol levels have been associated with ESR1 polymorphisms.

A long genotype group of a common thymine-adenine (TA) dinucleotide repeat polymorphism in the regulatory region of ESR1 has been associated with coronary artery disease. There is also possibility that carriers of the long repeat are marked by decreased expression of the ESR1 gene and that they have less cardiovascular protective effects from estrogen than carriers of the short alleles [8-10].

APOE alleles and genetic variants of IL-6 have also been established to be factors of susceptibility to obesity [1]. Data on C-174G polymorphism of IL-6 suggest that it affects the IL-6 transcription rate and plays a role in the development of obesity and insulin sensitivity [11].

LPL PvuII polymorphism is associated with high cholesterol levels and atherosclerotic cardiovascular diseases [12,13]. In our previous investigations ACE [14] and APOE [15-17] were confirmed to be metabolic risk factors.

In this study we tried to identify some genetic variables associated with lipid status and obesity in young healthy subjects.

## Methods

### Human subjects

A total of 105 young healthy subjects (58 female, 47 male), aged 20-35 years, participated in the study. They completed a questionnaire on family history of cardiovascular diseases, hypertension, obesity, taking medications, smoking and diet. All participants signed informed consent forms, and the study protocol was approved by Ethics Committee. BMI was calculated as weight (kg)/height (square meter), and WHR was calculated as the ratio of abdomen or waist to hip circumference. Blood samples for biochemical analyses (total cholesterol, LDL- and HDL-cholesterol, glucose, CRP, urate) were collected after overnight fasting and analyzed.

### DNA extraction and genotyping

#### ESR1-TA repeats

Dinucleotide polymorphism of ESR1 was analyzed using PCR amplification with labeled primer 5'-6-FAM-GAC GCA TGA TAT ACT TCA CC-3' and 5' - GCA GAA TCA AAT ATC CAG ATG-3'. PCR was performed with 25 cycles that consisted of 2 min at 94°C, 1 min at 58°C, and 1 min at 72°C, followed by 30 min at 60°C after the last cycle. The alleles were size-separated by capillary gel electrophoresis using Gene Scan Fragment Analysis Software 4.0. In short, 1 μl of the product was diluted with 12 μl of deionized formamide containing 0.5 μl GeneScan-500 ROX internal lane standard for sizing DNA fragments. Capillary electrophoresis was then carried out using ABI PRISM 310 Genetic Analyzer and POP-6 that had been formulated for applications requiring high resolution under denaturing electrophoretic conditions [8].

#### IL-6 G-174C

The primers used in the PCR were: 5' TGACTTCAGCTTTACTCTTTGT 3' and 5' CTGATTGGAAACCTTAT TAAG 3'. The reaction was carried out in a final volume of 50 μl containing 1.5 mmol/l of MgCl2, 0.2 mmol/l of each dNTP, 0.2 mmol/l of each primer, and 2.5 unit of Taq polymerase. DNA was amplified during 35 cycles with an initial denaturation of 10 minutes at 94°C and a final extension of 10 min at 72°C. The cycle program consisted of 1 minute denaturation at 94°C, 1 minute and 35 s annealing at 55°C and 1 min extension at 72°C. PCR products were digested with Nla III restriction enzyme at 37°C overnight and electrophoresed on a 2% agarose gel with ethidium bromide staining.

The identified genotypes were named according to the presence or absence of the enzyme restriction sites, so Nla III (GG), (GC), and (CC) are homozygotes for the presence of the site (140/58 bp), heterozygotes for the presence and absence of the site (198/140/58 bp), and homozygotes for the absence of the site (198 bp), respectively [11].

#### LPL P+/- and APOE epsilon2, epsilon3, epsilon4

LPL P+/- was determined using PCR-RFLP and agarose gel electrophoresis technique, as previously described in [13]

APOE genotyping was performed by applying DNA assay that uses rapid-cycle PCR and fluorescence resonance energy transfer (FRET) with the LightCycler. In the assay, a 265-bp fragment of exon 4 of the APOE gene was amplified from genomic DNA. The detection probes that cover codons 112 and 158 were 5' labeled with LC-Red 640 and LC-Red 705, respectively [18].

### Statistical analyses

Descriptive statistics were used to present all sociodemographic and clinical features of subjects. Data were given as means +/- standard deviations (SD) and frequencies/percents, as appropriate. Statistical analyses were performed to:

1) Study the associations of sociodemographics and all clinical features (Table 1) with metabolic parameters. The differences between mean values of metabolic parameters (BMI, abdominal obesity, cholesterol, LDL, HDL, triglycerides, glucose, blood pressure, CRP and urate) among different groups according to clinical features and sociodemographics were determined using t-tests for independent samples or analysis of variance, as appropriate. In case of significant differences between groups, logistic regression was performed to test for the predictors of BMI, abdominal obesity, high cholesterol, high LDL, low HDL, high triglycerides, glucose intolerance, hypertension, abnormal CRP and urate. For that purpose variables describing metabolic status (BMI, abdominal obesity, cholesterol, LDL, HDL, triglycerides, glucose, CRP and urate) were grouped in categories according to their normal values - described in Table 2.

**Table 1:** Sociodemographic and clinical data of subjects (N = 105)

	**Mean +/- SD**
Age (yrs)	25.82 +/-3.28
Weight (kg)	73.99+/-16.09
Height (cm)	175+/-10.53
BMI (kg/m^2^)	23.48+/-3.81
Waist (cm)	83.61+/-12.04
Hip (cm)	103.23+/-8.32
WHR	0.81 +/- 0.8
Triglycerides (mmol/L)	1.08+/-0.55
Cholesterol (mmol/L)	4.53+/-0.96
HDL-C (mmol/L)	1.45+/-0.3
LDL-C (mmol/L)	2.59+/-0.85
CRP (mg/L)	1.34+/-1.61
CRP (mg/L)	1.34+/-1.61
Glucose (mmol/L)	5.03+/-0.45
Urate (mmol/L)	292.70+/-76.35

**Table 2:** Clinical and metabolic parameters of subjects (N = 105)

**Feature**		**Frequency**	**Percent**
Somatic illness	No	78	74.3
	Cardiovascular illness	4	3.8
	Other illnesses	14	13.3
Smoking	Yes	67	63.8
	No	31	29.5
Medications	No	87	82.9
	With known effects on investigated features (Contraceptives)	9	8.6
	Other medications	4	3.8
Elevated hepatic enzymes	No	77	73.3
	Yes	19	18.1
Diet	Continental	75	71.4
	Mediterranean	13	12.4
	Mixed	10	9.5
Positive family history	No	25	23.8
	Diabetes	12	11.4
	Cardiovascular disease	32	30.5
	Obesity	2	1.9
	More than one	34	32.4
Hypertension (>135/85 mmHg)	No	79	75.2
	Yes	19	18.1
BMI (kg/m^2^)	<25	77	73.3
	25.01-30	23	21.9
	> 30.01	5	4.8
Abdominal obesity (WHR >90 (M), >85 (F)	No	85	83.3
	Yes	17	16.7
Hypertriglyceridemia (>1.7 mmol/L)	No	93	88.6
	Yes	12	11.4
Hypercholesterolemia (>5 mmol/L)	No	81	77.1
	Yes	24	22.9
HDL-C (<1.0 mmol/L)	No	98	93.3
	Yes	7	6.7
LDL-C (>3 mmol/L)	No	81	77.1
	Yes	24	22.9
Glucose levels (>6.4 mmol/L)	Hypoglycemia	4	3.8
	Normal	100	95.2
	Hyperglycemia	1	1.0
CRP levels (mg/L)	< 1	61	58.1
	1-3	34	32.4
	> 3	10	9.5
Elevated urate levels (>383 mmol/L)	No	93	88.6
	Yes	12	11.4

M - male; F - female; HDL-C - high density lipoprotein; LDL-C - low density lipoprotein cholesterol; CRP - C-reactive protein

2) Study the association of genetic variants with lipid status and BMI. For that purpose variables describing metabolic status (BMI, abdominal obesity, cholesterol, LDL, HDL, triglycerides, glucose, blood pressure, CRP and urate) were again grouped in categories as described in Table 2. Associations of alleles, genotypes and haplotypes with biological variables were established using UNPHASED-3.0.10. Sex and age were used as covariates [19,20].

The sequential Bonferroni adjustments (Holm, 1979; Rice, 1989) were applied to correct for the effect of multiple tests using SAS Release 8.02 (SAS Institute, 1999).

## Results

Basic characteristics of 105 study participants are shown in Table 1 and Table 2. Age of the subjects was 25.82 +/- 3.28 years. BMI values ranging between 25 - 30 were found in 21.9% of subjects. Positive family history for obesity was established in 32.4% of subjects.

Hypertriglyceridemia was detected in 11.4% of subjects. 12.4% of subjects were on Mediterranean diet. In addition, increased CRP levels were found in 9.5% of our subjects, and elevated urate levels in 11.4%. Out of 105 subjects, 9 were taking medication for cardiovascular diseases, 4 were taking other medications, for 5 subjects data were missing and the rest (N = 87) were not using medications at the time of the study.

We found an association between BMI and diet type. Differences in weight, BMI, waist measures, and WHR of subjects with regard to diet type (continental, Mediterranean, mixed type) were significant (p = 0.015; p = 0.023; p = 0.01; p = 0.015, respectively) (Table 3). Post hoc analysis showed that mixed type of diet contributed to significantly different weight, waist and BMI of subjects compared to those having the other two types of diet, whereas Mediterranean diet contributed to significantly different WHR compared to subjects on the other two types of diet (Table 3). However, logistic regression did not confirm diet type as a predictor of abdominal obesity and BMI (data not shown). The most favorable results in weight, BMI, WHR measurement were established for subjects on Mediterranean diet (as compared to continental and mixed diet): the type of diet, as an environmental factor, was found to have considerable impact on health of young population. Further, analysis of association between ESR1 genetic polymorphisms and APOE and hypercholesterolemia is presented in Table 4. In relation to hypercholesterolemia, a significant interaction was found for ESR1-L allele (p = 0.011; df = 1; X^2 ^= 6.391). Significant association was also established between APOE4 and hypercholesterolemia (p = 0.003; df = 2; X^2 ^= 11.46) (Table 4). Abdominal obesity was correlated to LPL P+/-, P+/+ genotypes (p = 0.013; df = 2; X^2 ^= 7.794) in a significant manner (Table 5). WHR (>0.9 -M, 0.85 - F) was found to be a good indicator or the risk for obesity among heterozygous and homozygous subjects for LPL genotype.

**Table 3:** Differences in weight, BMI, waist and WHR of subjects with regard to their diet type

		**Diet**		**ANOVA**,	**LSD post hoc**,
	**Continental**	**Mediterranean**	**Mixed**	**p value**	**p values (only significant)**
Weight (kg)	74+/14.56	66.61+/- 15.84	86+/-23.86	F = 4.365, p = 0.015	Mixed vs. continental, MD = 12.18, p = 0.025 Mixed vs. Mediterranean, MD = 19.58, p = 0.004
BMI (kg/m^2^)	23.5+/-3.21	21.73+/-2.85	26.15+/-7.31	F = 3.915, p = 0.023	Mixed vs. continental, MD = 2.65, p = 0.039 Mixed vs. Mediterranean, MD = 4.42, p = 0.006
Waist (cm)	83.65+/-11.04	76.77+/-10.83	92.1+/-17.37	F = 4.803, p = 0.01	Mixed vs. continental, MD = 8.45, p = 0.036 Mixed vs. Mediterranean, MD = 15.33, p = 0.003
WHR	0.81+/-0.07	0.76+/-0.08	0.86+/-0.09	F = 4.421, p = 0.015	Mediterranean vs. continental, MD = -0.05, p = 0.042 Mediterranean vs. mixed, MD = -0.095, p = 0.004

MD = mean difference

**Table 4:** Association of ESR1 and APOE genetic polymorphisms and hypercholesterolemia

			**Cholesterol < 5 mmol/L**	**Cholesterol > 5 mmol/L**	**OR**	**CI**	**Test p value**
ESR1*	Alleles	S	87	14	1	1-1	X^2 ^= 6.391
		L	77	30	2.421	1.242-4.72	df = 1
							p = 0.011
							
	Genotypes	SS	22	3	1	1-1	X^2 ^= 7.147
		LS	43	8	1.364	0.3288-5.661	df = 2
		LL	17	11	4.745	1.141-19.73	p = 0.028
							
APOE**	Alleles	ε2	21	1	1	1-1	X^2 ^= 11.46
		ε3	123	32	5.463	0.6664-44.79	df = 2
		ε4	14	11	16.5	1.366-199.2	p = 0.003
							
	Genotypes	22	2	0	1	1-1	X^2 ^= 14.98
		23	15	0	8.413e-006	8.413e-006-8.413e-006	df = 4 p = 0.004
		24	2	1	1.285e+008	2.495e+007-6.62e+008	
		33	48	11	5.891e+007	2.27e+007-1.529e+008	
		34	12	10	2.1424+0088	7.876e+007-5.825e+008	

APOE = apolipoprotein E

ESR1 = estrogen receptor alpha

* Means+/-SD of SS, LS and LL were 4.2+/-0.9; 4.56+/-0.97; 4.81+/-0.89, respectively

** Means+/-SD of 22, 23, 24, 33 and 34 were 3.65+/-0.92; 3.89+/-0.58; 3,83+/-1.44; 4,51+/-0.74; 4.51+/-0.95, respectively

**Table 5:** LPL genotyping results that indicate a risk associated with abdominal obesity

			**WHR** **<0.9 (M); <0.85 (F)**	**WHR** **>0.9 (M); >0.85 (F)**	**OR**	**CI**	**Test** **p value**
	Alleles	P-	90	18	1	1-1	X^2 ^= 1.158
		P+	68	22	0.6641	0.203-2.18	df = 1
LPL							p = 0.272
	Genotypes	P-/-	26	2	1	1-1	X^2 ^= 7.794
		P-/+	38	14	0.1057	0.01-0.86	df = 2
		P+/+	15	4	0.28	0.023-3.37	p = 0.013

LPL = lipoprotein lipase

OR = Odds ratio

CI = confidence interval

* Means+/-SD of P-/-, P-/+ and P+/+ were 0.83+/-0.07; 0.79+/-0.08; 0.79+/-0.08, respectively

Association between blood pressure and IL-6 CC genotype was not found to reach statistical significance (p = 0.069; df = 2; X^2 ^= 5.326) (Table 6). Nevertheless, it was substantial enough to indicate an important correlation between high blood pressure and IL-6 (CC-genotype), which should be examined on a larger study sample.

**Table 6:** Association of IL-6 genetic polymorphisms and blood pressure

			**BP < 135/85**	**BP > 135/85**	**OR**	**CI**	**Test** **p value**
IL-6	Alleles	C	95	20	1	1-1	X ^2 ^= 0.952 df = 1
		G	71	10	1.495	0.78-2.86	p = 0.329
							
	Genotypes	CC	30	9	1	1-1	X ^2 ^= 5.326
		CG	35	2	5.25	1.05-26.21	df = 2
		GG	18	4	1.35	0.363-5.03	p = 0.069

BP = blood pressure

OR = Odds ratio

CI = confidence interval

## Discussion

Genetic biomarkers have an increasing impact in laboratory medicine. They are currently used to predict disease onset or disease recurrence, and to individualize treatment and assess response to treatment.

We found an association between BMI and diet type. Differences in weight, BMI, waist measures, and WHR of subjects with regard to diet type (continental, Mediterranean, mixed type) were indicative. Subjects on Mediterranean diet had the best results with regard to BMI and WHR, which confirmed the assumption that the type of diet could have a substantial effect on the health of young subjects. Further, association was also established between ESR1 and APOE genetic polymorphisms and hypercholesterolemia. Actually, our finding was confirmed by results of other studies [1] who found positive correlation between APOE polymorphism and hypercholesterolemia. Association between estrogen receptor-alpha gene polymorphisms and coronary artery disease with familial hypercholesterolemia was previously reported in other studies [21].

Complex approach includes multiple genetic markers, gene expression, and functional protein. Such complex approach is needed for understanding physiopathology and etiology of metabolic disorders and cardiovascular disease, and leads to development of biomarkers for prevention of disease. Possible metabolic biomarkers that we examined in our study are CRP and urate: we found elevated levels (9.5%; 11.4%, respectively) of these metabolic parameters, and similar results in young adults were reported previously [22,23]. Recent data also suggest that elevated serum CRP and urate levels may contribute to the development of coronary artery disease [24]. It has been established that men with long alleles at the ESR1 promoter TA repeat have significantly more narrowed coronaries, larger areas of complicated lesions, and more calcification of coronary arteries than men with short alleles. A recent Finnish study proposed that long-repeat carriers have lower expression of the ESR1 gene and less cardiovascular protective effects from estrogen than carriers of short alleles [8].

In our study, we found an association between ESR1 long TA allele and hypercholesterolemia in young healthy subjects and it could represent a possible risk factor for developing metabolic disorder and cardiovascular disease.

For genetic prediction of disease risk, it is important to identify functional gene polymorphism in order to establish genotype-phenotype correlation. Polymorphisms can be associated with either health (low risk predictors) or possible development of disease (high risk predictors; hypercholesterolemia, hypertension, obesity, metabolic syndrome, cardiovascular disease). Adipogenesis-related genes such as PPAR gamma and genes related to cytokines such as IL-6 and lipid metabolism including LPL have also been associated to the weight lowering outcome induced by hypocaloric diets [25]. Efforts to reduce cholesterol level in order to prevent coronary heart disease have led to declined mortality. However, new information in this regard have recently been recorded in western countries: a decreasing trend in HDL cholesterol and increasing trends in obesity and triglycerides [26].

In literature, correlation between LPL polymorphisms and obesity has remained controversial; however, we found evidence of this association in our study. Actually, presence of LPL P+/-, P+/+ genotypes that we investigated in our study might, as a predictive risk factor related to obesity-related metabolic disorder, indicate the need for individualized diet adjustments.

A study reported previously [27] evaluated the role of IL-6-174G>C polymorphism in the risk of developing metabolic alterations in people with excessive body weight. Their data demonstrated that the occurrence of C allele of IL-6-174G>C gene polymorphism in people with excessive body weight was accompanied by increased risk of developing obesity-related metabolic disorders, especially insulin resistance.

Regardless of age and gender, subjects with IL-6 C and PPAR gamma variants had lower BMI and plasma triglyceride levels than those carrying only one of these variants. Thus, it might be possible that IL-6 gene polymorphism alone has none or only slight effects; its effects may be apparent only in the presence of a factor like PPAR gamma [4]. The influence of interleukin-6 G-174C gene polymorphism on coronary artery disease, cardiovascular complications and mortality in dialysis patients was described in a previously reported study. Their results suggest that IL-6 gene G-174C polymorphism is associated with the incidence of cardiovascular events and mortality in chronic dialysis patients [28]. Although this indicates an important correlation between high blood pressure and IL-6 genotype, larger studies are needed to confirm this significance.

## Conclusion

Results of examining correlation between weight, BMI and WHR and continental, Mediterranean and mixed diet showed optimal values in subjects on Mediterranean diet.

Statistically significant correlation was detected between ESR1 long allele and total cholesterol. We also found statistically significant associations of APOE 2/3 and APOE3/4 genotypes with lowered and increased cholesterol levels, respectively. Statistically significant association was observed between LPL P+/- polymorphism and abdominal obesity. These three polymorphisms indicate an association with traits that characterize lipid status and obesity.

## Competing interests

The authors declare that they have no competing interests.

## Authors' contributions

JS is a coordinator of the project involved in genotyping data quality control, interpretation and finalizing the manuscript; MM, ZR and BJ helped in conceptualizing the study and provided intellectual input; LJ and HL carried out molecular genetic studies and sample collection; TB participated in statistical analyses and data interpretation; JM and JL participated in manuscript drafting and data interpretation. All authors have read and approved the final manuscript.
